# Political economy analysis of sub-national health sector planning and budgeting: A case study of three counties in Kenya

**DOI:** 10.1371/journal.pgph.0001401

**Published:** 2023-01-04

**Authors:** Benjamin Tsofa, Evelyn Waweru, Joshua Munywoki, Khaing Soe, Daniela C. Rodriguez, Adam D. Koon

**Affiliations:** 1 KEMRI-Wellcome Trust Research Programme–KEMRI Centre for Geographic Medicine Research -Coast, Kilifi, Kenya; 2 Department of Public Health–Pwani University School of Health Sciences, Kilifi, Kenya; 3 United Nations Children’s Fund (UNICEF) Kenya, Country Office, Kisumu, Kenya; 4 Johns Hopkins Bloomberg School of Public Health, Dept. of International Health, Baltimore, Maryland, United States of America; University of Ghana College of Health Sciences, GHANA

## Abstract

Devolution represented a concerted attempt to bring decision making closer to service delivery in Kenya, including within the health sector. This transformation created county governments with independent executive (responsible for implementing) and legislative (responsible for agenda-setting) arms. These new arrangements have undergone several growing pains that complicate management practices, such as planning and budgeting. Relatively little is known, however, about how these functions have evolved and varied sub-nationally. We conducted a problem-driven political economy analysis to better understand how these planning and budgeting processes are structured, enacted, and subject to change, in three counties. Key informant interviews (n = 32) were conducted with purposively selected participants in Garissa, Kisumu, and Turkana Counties; and national level in 2021, with participants drawn from a wide range of stakeholders involved in health sector planning and budgeting. We found that while devolution has greatly expanded participation in sub-national health management, it has also complicated and politicized decision-making. In this way, county governments now have the authority to allocate resources based on the preferences of their constituents, but at the expense of efficiency. Moreover, budgets are often not aligned with priority-setting processes and are frequently undermined by disbursements delays from national treasury, inconsistent supply chains, and administrative capacity constraints. In conclusion, while devolution has greatly transformed sub-national health management in Kenya with longer-term potential for greater accountability and health equity, short-to-medium term challenges persist in developing efficient systems for engaging a diverse array of stakeholders in planning and budgeting processes. Redressing management capacity challenges between and within counties is essential to ensure that the Kenya health system is responsive to local communities and aligned with the progressive aspirations of its universal health coverage movement.

## Introduction

Public sector decentralisation which involves the transfer of decision-making from a national level entity to sub-national level entity continues to be a widely adopted public health sector governance reform in many Low- and Middle-Income Counties (LMICs). Various forms of decentralisation including devolution, deconcentration, delegation and privatisation have been documented across various setting. Nevertheless, irrespective of the form, the effects and outcomes of these different forms of decentralisation continues to be varied. The Kenyan health system has undergone multiple decentralization reforms over the year including some form of decocentration delegation and mor recently devolution [1, 2]. These have largely focused on transferring decision making over use of health sector resources to lower level management units, with appropriate oversight by governance structures including Facility Management Committees (FMCs) and District/Hospital Management Boards (D/HMBs) made of a mixture of elected community representatives, and appointed public officers [1, 3–7].

In the 90s, the first National Health Policy was developed, and was designed to be implemented through two five-year National Health Sector Strategic Plans (NHSSPs). Their implementation relied on a deliberative multi-level process of developing one-year Annual Operational Plans (AOPs) that, while imperfect, provided a platform for expanded engagement of multiple stakeholders in sub-national planning and budgeting [4, 6, 8]. This process of formalized health sector planning and budgeting was further strengthened when the Government of Kenya (GoK) adopted the Medium Term Expenditure Framework (MTEF) as a guiding framework for guiding public sector planning and budgeting in the country [4, 8–11].

In 2010, the country enacted the new Constitution of Kenya (CoK) that introduced political and fiscal devolution of some government functions to 47 semi-autonomous county governments [12]. Under the devolved government system, health service delivery function was assigned to county governments. The county governments receive a nonconditional *equitable share* of funding from the national government based on a Resource Allocation Criteria (RAC) established in law. The CoK 2010 has placed this funding to be a minimum of 15% of total national government revenue. Consequently, resource allocation for all investments required for routine health service delivery has been a mandate of county governments since 2013 [8, 12].

As outlined in the CoK 2010 and other subsidiary legislations, county governments are made of two independent arms. The first arm is the County Executive comprising of an elected Governor and their deputy, with a ten-member ‘county cabinet’ dubbed ‘County Executive Committee (CEC)”, whose members are responsible for one service delivery department within a county government. The County Department of Health (CDoH) is one of these ten service delivery departments. The Executive Arm is primarily responsible for implementing all government services that were assigned to counties by the constitution. The second arm is the County Assembly (CA), which consists of elected Members of the County Assembly (MCAs). Each MCA represents an electoral ward, although the CA has a few nominated members that represent special interests’ groups such as women, youths and people living with disabilities. CA slots for nominated MCAs are distributed across political parties based on the numerical number of each political party in the CA; more popular political parties get more slots for nominating MCAs [4, 8, 12].

Studies prior to the 2010 devolution reported that health sector planning and budgeting process was often top-down, with minimal inputs and participation by communities, facility and district managers [8, 13, 14]. In addition, a consistent challenge of misalignment between the planning and budgeting processes was often reported [1, 3, 4]. More recent (post-devolution) studies have reported capacity challenges among county-level managers that are responsible for health sector planning and budgeting, lack of formal criteria for guiding county-level health sector priority setting, and over politicization of the health sector priority setting processes at the county level [8, 9, 11, 15].

Besides *equitable share* of revenue allocated to counties, the CoK allows the national government to provide additional conditional resources to counties to support implementation of service delivery and development projects that are a priority of the national government–including health sector projects. Since 2013, the national government has funded county governments to implement national government agendas such as removal of user fees for primary health facilities, provision of free maternal health services, and more recently, rolling out of universal health coverage (UHC)-driven initiatives that are part of Kenya’s *‘Big 4 Agenda*. [6, 16, 17] The national UHC rollout was first piloted in four counties namely Machakos, Nyeri, Isiolo and Kisumu counties in 2018, after which national scale up began in 2020 [18]. Table 1 below summarizes key health sector reforms that have influenced sub-national health sector governance in Kenya since 1994 [19].

**Table 1:** Summary of key sub-national health sector governance reforms in Kenya.

	Health Sector Reform	Year
1	Introduction of user fees in public health facilities and establishment of District/Hospital Management Boards	1989
2	Development of the Kenya Health Policy Framework	1994
3	Development of the Second National Health Sector Strategic Plan, and Introduction of Annual Operational Planning	2005
4	Introduction of the Health Sector Services Fund	2010
5	Adoption of the Constitution of Kenya 2010	2010
6	Enactment of the Public Finance Management (PFM) Act 2012	2012
7	Enactment of the County Government Act 2012	2012
8	Establishment of 47 Semi-Autonomous County Governments	2013
9	Introduction of the Government Big 4 Agenda, and national UHC rollout	2018

Political economy analysis (PEA) is an analytical tool useful for understanding the incentives, power dynamics, behaviours, and constraints in a system, and how these influence resource distribution and policy and programmatic decisions over time. Though there is no universal definition or framework for conducting PEA, core elements include interaction of political and economic processes, relationships and power dynamics between stakeholders and how these change over time. In the context of health systems and policy analysis, PEA can help to understand how ideas, interests, and formal and informal institutions influence, and are influenced by, decision-making, power and resources thus shaping political action and the implementation of health policies.

The United Nations Children Fund (UNICEF) has been supporting the Ministry of Health (MoH) Kenya in various ways, one of which is through supporting the implementation of the District Health Systems Strengthening Initiative (DHSSi) in Kenya, Uganda and Malawi [20]. DHSSi is implemented at the subnational-level and aims to strengthen the capacity for health sector management around priority setting, planning, and budgeting. In Kenya, the DHSSi is being implemented in 5 counties namely: Garissa, Isiolo, Kisumu, Siaya and Turkana. These counties were selected as part of a broader UNICEF program of work agenda for supporting counties with poor child health outcomes. At the start, DHSSi identified management gaps and challenges that are a barrier to good management practices across all the three countries. Bottlenecks identified included: poor use of available evidence for decision-making, difficult power relationships between national and sub-national levels, complex governance arrangements at sub-national level that had overlapping roles among health sector managers, and poor coordination of development partner activities. Owing to this, UNICEF requested for a political economy analysis (PEA) of how a sub-national-level decision-making environment affects priority setting, planning, and budgeting for health across the three countries. Thus, the overall aim of this study was to undertake a PEA of sub-national level health sector governance, with a focus on health sector priority setting, planning and budgeting.

This paper reports on the findings from this PEA in Kenya. By applying political economy analysis, our study adds to the growing body of literature that has studied health sector priority setting, planning, and budgeting. We believe that this provides more concise problem identification and problem-solving ideas into the health sector priority setting, planning, and budgeting strengthening agenda.

## Methods

**Fig 1:**
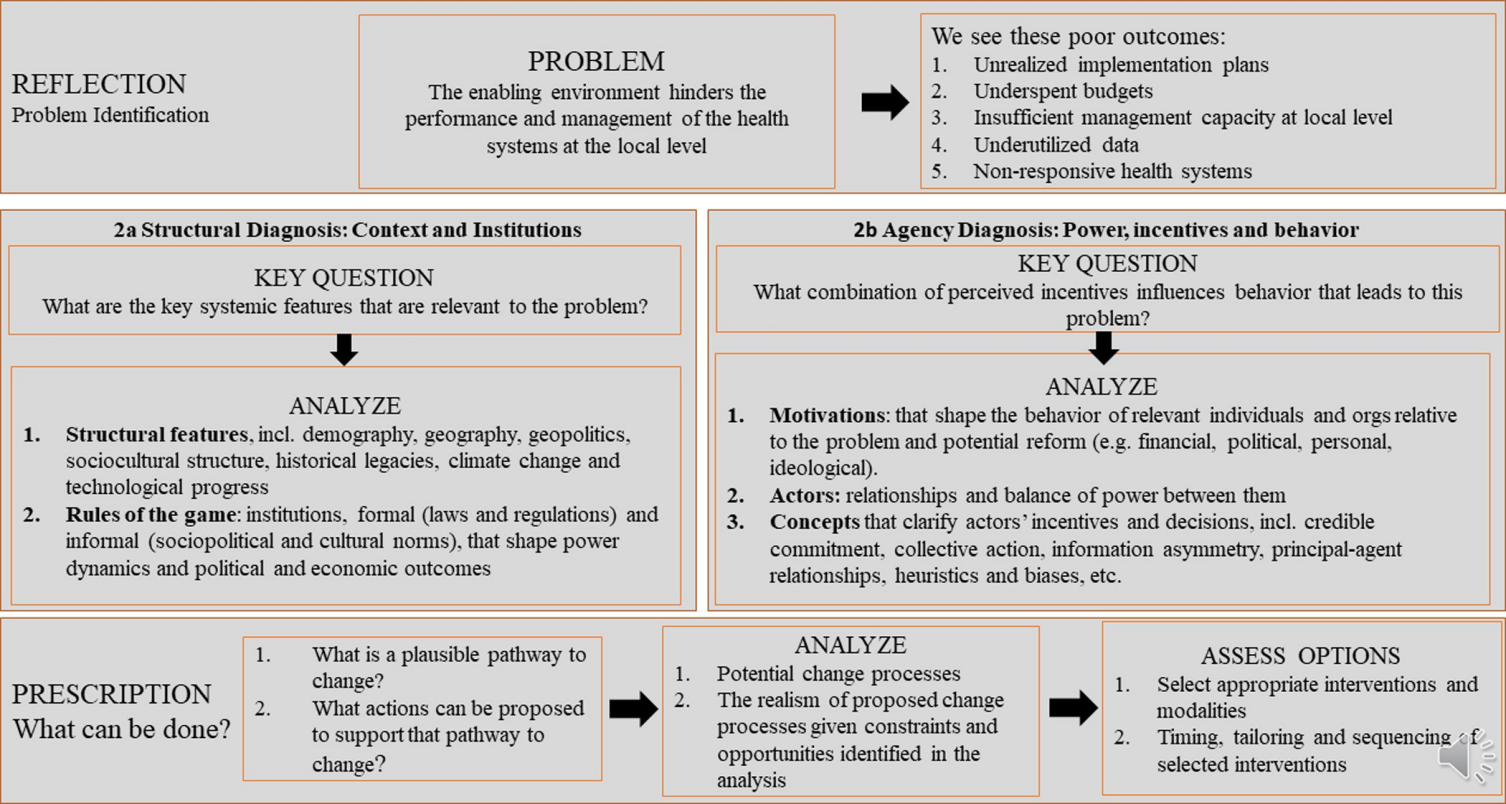
Problem-driven PEA theoretical framework.

We used a problem-driven PEA approach, which focuses on a specific problem or policy—as opposed to a whole country or sector—to better understand a challenging issue, the institutional dynamics contributing to the problem, and the broader actors and systems factors that facilitate or hinder change [21–23]. For this study in Kenya, we focussed on county-level health sector priority setting, planning, and budgeting as the problem/policy issue. A critical feature of problem-oriented PEA is its operational, practice-oriented nature that lends itself more readily to generating practicable, politically realistic recommendations that consider the risks of taking action [22, 23]. Fig 1 illustrates the analytical pathway for the problem-driven PEA, as adapted from Siddiqi et al., 2009 [24]. From our framework, structural diagnosis focusses on *structural features* and *rules of the game* that oversee, coordinate, and facilitate a relevant policy issue. Agency diagnosis focuses on *actor*s, *motivations*, and *concepts* around a policy issue. And finally change focuses on *implementation* experiences and *pathways to change* and/or improvements of the policy issue. We structured our interview guides around these three broad thematic areas of the PEA framework. In addition, since we undertook our data collection about a year into the Corona Virus Disease (COVID19) pandemic, we added on our data collection tools some additional questions and probes to understand if and how the COVID19 outbreak had affected the health sector planning and budgeting processes in the counties.

We applied this framework in developing our study data collection tools, during analysis by guiding the development of our initial coding frame and in discussing our study findings.

### Case selection

We used a phased approach to select 3 (Garissa, Kisumu and Turkana) case study counties for our study. The first phase of the study involved a review of secondary data and records from all the 5 DHSSi counties alongside consultation with national level stakeholders involved in county level health sector planning and budgeting. Guided by the study conceptual framework, we identified, accessed, and reviewed key records and documents with relevant information regarding county-level health sector planning and budgeting. These included:

1. Government documents related to devolution: the 2010 Constitution of Kenya, the County Government Act 2012, the Public Finance Management Act 2012
2. Health policy documents: the Kenya Health Policy (KHP) 2014–2030, the Kenya Health Sector Strategic Plan (KHSSP), the Health Sector Planning and Budgeting Tools and Guidelines
3. County documents: County Integrated Development Plans (CIDP), Annual Work Plans (AWP), health sector working group reports, medium-term expenditure framework (MTEF) guidelines
4. Research articles and reports related to the functioning of county governments.

Findings from records and documents review then informed selection of 3 study counties for phase 2. In selecting the 3 counties, we focused on allowing similarities and differences (as had been identified in the phase one analysis) to emerge for each individual case to support analytical generalizability of results. Specifically, the final section of the three counties factored in geographical location, rural/urban classification, adaptation of devolution guidelines, and implementation of DHSSi at county level.

### Data collection

In Phase 2, we collected qualitative data from the three case counties identified as described in Phase 1 above. We conducted key informant interviews (KIIs) (n = 32) with government officials, implementing partners, and health workers across all levels of the county health system, and with a particular emphasis on those involved in health sector planning, budgeting, implementation, and monitoring at the county level, and at the national level. Owing to the national COVID-19 prevention protocols that were in place at the time, interviews were conducted remotely either on an online platform or by telephone depending on network availability. A summary of respondent characteristics is included in Table 2.

**Table 2:** Characteristics of interview respondents.

Participants Category	County A (Garissa)	County B (Kisumu)	County C (Turkana)	National
County Level Health Managers	3	4	3	-
County Level Treasury	1	0	1	-
County Level Health Partners	1	0	0	-
Sub-County Health Managers	2	4	2	-
Facility Health Managers	2	3	1	
Facility Management Committee/Member of County Assembly	1	0	1	-
National Level Participants	-	-	-	3
**Totals**	**10**	**11**	**8**	**3**

### Data analysis

We utilized a framework analysis approach to analyze the data. All interviews were audio recorded and transcribed verbatim. This was followed by a review of transcripts, during which data from the transcripts were summarized and abstracted to a matrix in Microsoft Excel that contained themes and sub-themes that we developed from the elements and sub-elements of the PEA conceptual framework. In the abstraction matrix, each row represented an interview respondent whereas each column represented a PEA theme or sub-theme.

To enhance rigor and validity of the findings, we employed a range of strategies including, triangulation, member checking, and fair dealing. Triangulation was pursued using multiple data sources including document reviews, interviews, while cross-checking for consistency. We ensured reflexivity by conducting regular research team reflection meetings, critically appraising how our own individual and collective positionality, values, beliefs and experiences; and how the study design and conduct of the research (and especially the remote interviews) could influence data collection, analysis and interpretation of findings and conclusions. We crosschecked preliminary data and initial analysis themes for validation with some of the key informants. In the identification and selection of key informants, we ensured that we consciously incorporate a wide and broad base of participants to ensure coverage of a wide range of views and perspectives of respondents.

We conducted two virtual feedback meetings 1) with national level stakeholders and 2) UNICEF zonal officers involved in supporting the implementation of the DHSSi project in Kenya. In addition, we presented the preliminary findings to an internal audience of Health Policy and Systems Research colleagues at the KEMRI-Wellcome Trust Research Programme in Kenya to get further inputs and feedback on our interpretation of the findings.

### Ethical considerations

Ethical review and approval for this work was obtained from the African Medical and Research Foundation (AMREF)–Kenya, Ethical Review Committee (ref: AMREF ESRC-P855/2020, and a research permit obtained from the National Council for Science, Technology, and Innovation (Ref No: 785446). Potential participants were provided with comprehensive information sheet describing the study together with a written consent form prior to conducting a KII. At the end of an interview session study participants were reimbursed KES 500 or KES 1,000 as reimbursement of airtime and/or internet data cost based on participants reported estimated costs of data. These reimbursements rates were approved by the ERC. To maintain confidentiality, prior to analysis, we removed all personal identifiers of the participants from the audio tapes, transcripts, and analysed reports. In addition, we anonymized the study counties by assigning each county an identity code.

## Results

In this section, we begin by presenting our findings on the actors involved in county-level health sector planning and budgeting in Kenya. We then explain the process and coordination of county level health sector planning and budgeting in the case study counties. We further highlight the drivers and influences of health sector priority setting in these counties. We proceed to highlight the accountability structures and practices in these counties. We then present findings on the challenges affecting health sector planning and budgeting and highlight some of the outcomes of the process in the case study counties. We conclude by highlighting how the county health sector planning and budgeting was affected by the COVID-19 pandemic.

### Actors involved in county health sector priority setting, planning, and budgeting

#### Actors involved and their roles

Devolution increased the number of actors involved in health sector planning and budgeting both at national and county level. Table 3 outlines all the key actors at both levels, who have had a decision-making role during the county-level resource allocation, planning and budgeting for health. It goes further to illustrate the respective roles of these actors.

**Table 3:** Actors involved in national and sub-national health management.

Level	Actor	Roles
National	National Treasury	• Manages and coordinates overall government budgeting process • Provides guidelines on process and timelines and annual strategic priorities through circulars
	Ministry of Health	• Coordinates sectoral planning, budgeting process • Develops planning tools and guidelines for guiding county health sector planning • Capacity building for county planning and budgeting • Coordinates and monitors implementation of national level conditional grants/projects (e.g. Linda mama, UHC roll out, etc.) to counties
	Council of Governors Secretariat	• Act as liaison between national government (MoH/Treasury) and the counties • Involved in dissemination of national policies and guidelines to counties and coordinating county level capacity building by national government
	Development partners	• Support MoH activities/initiatives • Capacity building for national, county government • Advocacy
	Parliament	• Final approval of national government budgets
County	County Assembly/ Members of County Assembly	• Final approval of health sector plans and budgets • Amends/reprioritizes budgetary allocations after review by SWGs and Directorate of Planning before they approve a budget • Represent community interests during resource allocation process
	County Executive Committee	• Approval of county plans and budgets before presentation to the county assembly
	(Health) Sector Working Groups	• Undertakes consolidation of health sector priorities within the county, as subset of the CHSF
	County Health Stakeholders Forum (CHSF)	• Made of all health sector implementing and funding partners in the county • Supports CDoH/CHMT priorities and initiatives
	County Department of Health (CDoH)/ County Health Management Team (CHMT)	• Provides data and evidence during the planning • Coordinates the technical priority identifications and planning • Lower levels of management (facilities and sub-counties) are involved in planning process but discretion over CDoH priorities and CDoH resource utilization rests with the County Health Management Team (CHMT) members such as the County Executive Committee member and Chief Officer
Community	Facility Management Committees (FMCs) and Public Forums	• Starts bottom-up development of plans & budgets • Reviews budget proposals and plans in public fora • Exercise power for demanding accountability both directly and through representatives e.g. facility management committee (FMCs), facility boards and Members of County Assembly.

#### Coordination of actors and stakeholders

Health sector stakeholder involvement in health sector planning and budgeting process is outlined in the Kenya Health Sector Partnership Framework (KHSPF) 2018–2030. At the national level, donors and partners are engaged by MoH units and departments through the Inter Agency Coordinating Committees (ICCs). It is at the ICC where donors make their budget support commitments, which are subsequently captured in the MoH MTEF. The MTEF forms the basis of the rolling MoH annual work plans and budgets at the national level.

KHSPF 2018–2030 has outlined the creation of County Health Sector Stakeholders Forum (CHSSF), while the Public Finance Management Act 2012 outlines the creation of Sector Working Groups (SWGs) as the mechanisms for engaging local donors and implementing partners in sector resource allocation, planning and budgeting. To be compliant and in sync with the PFMA 2012, the KHSPF 2018–2030 further states that the health sector SWGs membership (whose number is restricted by provisions of the PFMA 2012) should be selected through the CHSSFs [25].

*“From the ministry perspective*, *…now there is a partnership framework* (a policy document- the KHSPF 2018–2030) *that sets out how partners can work together with the ministry and the counties through a more organised institutional arrangement…” D P 2*, *National Level*

However, across all the case study counties, we found that the CHSSFs were not fully functional. Consequently, all SWGs members including health were appointed by the respective Chief Officers for Treasury. Interviewees reported that donor and other stakeholders’ engagement for health sector planning and budgeting in the counties happened in an ad-hoc fashion.

#### Community and public involvement and participation

Both the devolution laws and the PFMA 2012 have outlined various mechanisms for community engagement and public participation in planning and budgeting. These mechanisms include direct participation by community members through open public forums/meetings, and indirectly through community representatives such as Facility Health Management Committee members (FMCs), and elected MCAs at the assembly.

In practice though, the functioning of these mechanisms varied from county to county. Interviewees reported that open public hearings were often done in a hurry “*just to tick a box*” and due to this, they were often poorly attended and/or did not have representation from most of the community segments.

*“So because the law requires them (*County treasury*) to do public consultation for the government budget… eehh they normally organize the public forums in a hurry*. *Because of this the meetings are poorly attended*. *Then they (*treasury*) say we have done public participation*. *Yet you can see its just to tick a box*. *There is no genuine public participation during the budget making*.*”* County Manager 10, County B

Some respondents also observed that some of the facilities in the counties either lacked or had dormant FMCs; funds meant to support FMC activities either delayed or did not come and this discouraged FMC members. Public participation through the County Assembly was reported as the most consistent and active across all case study counties. This was said to have been so because of the more power that elected MCAs have, and the fact that they are consistently resourced to facilitate their roles and activities, hence were motivated to do so.

Where it happened, public participation often revealed a conflict of interest between the various actors involved. For instance, in case county B, CDoH managers felt that the public should just be given health awareness but not necessarily be involved in planning and budgeting for health.

#### Relationships and power dynamics between actors

Across all the counties, respondents reported existence of tense relations between the two arms of county governments during budget making and priority setting. These tense relations were attributed to the differences in interests and power dynamics between the executive and the legislative arm s of the county government during the resource allocation and budgeting process. Tense relations were also reported between different units and divisions within the CDoH as they bid to compete for the limited resources available during the budgeting process.

*“The assembly represents the people*, *the executive represents government*. *Yeah*, *just to agree that this and this will not be done in this financial year*, *that is the biggest issue*.*”* County Manager 3, County C

*“…you see for government there is a lot of things that come into play like politics*. *And you see each and every directorate wants to be seen as they are the ones working*. *So you might find that there can be some conflicts*‥*”* County Manager 5, County C

Across all the three counties, the relationship between community members and the CDoH was described to be strong. This was partly attributed to the involvement of FMCs in day-to-day decision making at health facilities–especially those that had functioning FMCs.

### Process and coordination of health sector planning and budgeting

All three counties reported having a functional directorate responsible for policy, planning, budgeting, monitoring and evaluation within the CDoH. These directorates acted as the coordinating arm of the health sector SWGs. The planning and budgeting process was similar in all the case study counties and was generally in line with the procedures outlined in the CoK 2010, the PFM Act 2012, and the subsidiary devolution laws. Respondents reported that the planning and budgeting process began with a circular from the National Treasury that gives timelines for planning and budgeting activities. Once this circular is received at the county level, the County Treasury issues its circular to the various county departments to kick off the planning process. Within the CDoHs, the CHMTs organize sensitizations for the various managers of planning units on how to undertake planning and budgeting.

From the county level KIIs, the planning and budgeting process in all the case study counties was reported to be happening in a bottom-up manner. Lower primary facilities present their plans and budgetary needs to the sub-county, where rationalization and consolidation of facility needs is done. The sub-county then submits both facility needs and sub-county coordination and administration costs to the county level. The county level then consolidates what has been submitted from the sub-counties and includes county-level needs as well. Rationalization at the county level factors county-level priorities as captured in the County Integrated Development Plan (CIDP). As the bottom-up planning and budgeting process reaches finalization at the county level, the County Treasury then issues budget ceilings to all county departments–including the CDoH. It is at this point that the County Treasury convenes SWG–including health–to determine, from the consolidated and rationalized CDoH plans and budgets, what to prioritize in a particular year.

In undertaking the planning and budget process, interview respondents reported that the CHMTs endeavour to engage with and involve multiple stakeholders. From the interviews with facility managers across the study counties, they reported that they were always involved in the process, but their inputs were more of a ‘wish list’ as they were rarely captured in the final CDoH budget. This led to low motivation in their participation; those who participated in planning and budgeting reported to be doing so ‘just to fulfil a routine requirement’.

*“And I think this (planning and budgeting) is one of the key areas that is largely being planned within the county government just because you know*, *it’s a process that requires to be implemented*. *But at the end of the day*, *whether it is implemented or not people dont get the resources that they required…”* County Manager 1, County A

#### Influences and incentives driving priority setting during health sector planning under devolution

Participants across the case study counties reported that health sector priority setting during planning and budgeting was driven by various influences and incentives. These included national priorities, county-level political interests, equity considerations, and relationship dynamics among actors involved in planning and budgeting.

#### National priority agenda

The 2017–2022 national government’s *Big 4 Agenda* has outlined UHC implementation as a priority, and this had a significant influence on health sector resource allocation and budgeting. Case study county B, for example, was among the initial 4 pilot counties that received additional health resources allocated from the national government for UHC roll out. As part of this arrangement, county B received additional medical commodities from the national government through the Kenya Medical Supplies Agency (KEMSA). The county (as was with the other pilot counties under this arrangement) was in turn required to remove user fees from all public health facilities. However, interviewees from this county reported that this arrangement with the national government did not work very well as the order-fill-rate they received for commodities from KEMSA was always not optimal.

*“*‥*For instance in 2018 when the UHC pilot was launched*. *And you know how the pilot was launched was that seventy percent of the funds were wired directly to KEMSA to provide health products and technologies …*.*in that budgeting year the MCAs opted not to put any allocation under pharmacy…the argument was that no*, *now we have–a lot of money have been put in KEMSA by the national government*, *… So the vote-head for commodities was zero in that year*.*”* County Manager 1, County B

#### Local/County level political considerations

At the county level, political considerations such as the governor’s manifesto and political promises by locally elected MCAs were said to strongly influence priority setting during the health sector planning and budgeting process. Respondents reported that decision making around resource allocations had to balance between both technical needs of CDoH and political needs of elected political leaders. Consequently, all the counties seemed to prioritize infrastructure projects because *‘development projects’* were more visible than other priority agendas. The MCAs were also reported to be benefiting directly from these infrastructure projects by having an influence over who to award contracts for these development projects. These local level political considerations led to a massive increase in construction of new health facilities as seen in county C, and construction of a cancer treatment centre in county A.

*“You see a budget is a political tool so before you see that budget being an Act*, *we have undergone a lot of pressure*, *there is a lot of politicking*. *So I see it’s like an achievement when we’ve passed our budget…you might have a very good solution but because it is not working for them political your views might not be considered*.*”* County Manager 5, County C

#### Equity considerations

All the case study counties reported to actively make efforts that enhance equity during planning and budgeting. Equity considerations was regarded as deliberate efforts to allocate resources to provide series to marginalised communities within the county, even if such services cost more. For instance, while developing their budget proposal, case study county C once allocated resources for an army truck (belonging to an army barracks within the county) to serve as a mobile clinic for nomadic populations residing in the county. In case study county A, it was reported that clan representation during county government recruitment and appointments was a key consideration.

*“So what is usually done*, *every family or every clan is given a slot (during county government recruitments)*, *so everybody is trying to in a way to advance the interest of the community or his family member in one way or the other*.*” County manager 5*, *County A*

#### Relationships of actors involved in the process

Across the counties, interviewees found relationships among actors involved in the resource allocation, planning and budgeting process to influence prioritization of resources. In county C, for example, the CDoH had difficulties in convincing the County Assembly to allocate resources to upgrade the county referral hospital. The County Director of Medical Services took an initiative to engage and mobilize several health sector stakeholders to a stakeholders meeting to deliberate of this. He illustrated to them the value and benefits of upgrading the county referral hospital. The mobilized stakeholders then joined with CDoH in the agitation for upgrading of the hospital. This finally forced the County Assembly to agree to allocate additional resources to the hospital for an upgrade.

### Accountability practices for county level health sector planning and budgeting

We found devolution to have increased accountability of the CDoH and the health system in general to the community. For instance, community members can now demand accountability from health facility in-charges through community representatives that sit in the FMCs and hospital boards. FMCs and hospital boards are also able to follow up on delayed resources with senior county government executives and other relevant county level leaders. Additionally, community members have been able to follow up on broader health sector issues with their respective MCAs. For instance in case county C, it was reported that communities would refuse to be associated with any development project that they had not determined as a priority during public participation.

*Yeah*, *so when they {Community}decide this is what we want*, *you cannot come again with other things*. *Yeah*, *they say*, *“This is what we want*, *this is the person we want*, *this is…yeah*.*” And once they’ve said they’ve said*. *You cannot change their minds*. *And if you insist (and) you implement a project that the community did not want*, *they will just look at it like that*. *They will be coming and ask*, *whose project is this*? *It is for that person*, *not ours…*.*”* County Manager 3, County C

Other forms of accountability reported to be routinely in place included: periodic health facility performance review meetings, supportive supervision by county and sub-county managers to health facilities, monthly/quarterly/biannual performance reporting, internal and external audits, and treasury budget officers crosschecking a county department’s vote book before approving it to draw from its budgets.

*“…let’s say they {health facility managers} want to execute that–or they want to expend that money … they have to come to our office*, *they come with a requisition letter to seek that money for instance*. *So we have to check where there is money in their vote*. *We normally depend on the vote books…so we check where there is funds available for such specific period*. *We have to approve*, *it has to go to the procurement again…”* County Manager 3, County A

### Challenges affecting county health sector priority setting, planning, and budgeting

Interview participants across the counties reported various challenges that affected both development and implementation of county plans and budget.

#### Rushed planning process

From the interviews, various participants across all the three counties reported that within the health sector, the planning process was often done in a rush and inputs from the bottom-up approach did not get factored in the final CDoH budgets. Further, interviewees noted that the CDoH budgeting process relied heavily on historical/previous year expenditure, thus the budget did not significantly change from year to year and did not thus align to changing priorities each year.

*“If the ceiling would be brought to us earlier we would then plan as per the ceiling not as per the wish list so that then the people that we are supervising would not get demoralized and demotivated…[by] doing the same thing over and over and they are not getting a change*.*”* County Manager 6, County C

#### Insufficient budget ceilings

Across the study counties, budget ceilings set by the County Treasury during the budgeting process always fell way below the ‘wish list’ of the CDoH. The budget ceiling would force the CDoH to assign a small team of the CHMT to undertake a preliminary rationalization role of the priorities so that they can fit within the ceiling. The CDoH interview participants felt that budgetary ceilings limited how much the CDoH could plan and budget for. It was reported that final prioritization for the department’s needs was often determined by the SWGs and further modified by County Treasury and County Assembly during the approval process. For these reasons, there was a reported lack of enthusiasm for participating in the planning and budgeting process by health managers across the various levels.

*But time after time now they are saying that you keep on making us do this and that but what we get in return is usually–so they have now labeled this thing as an exercise in futility*. County Manager 6, County C

#### Erratic disbursements of funds

During the budget execution phase, a key challenge reported across all counties was the erratic release of allocated funds by National Treasury to the county governments. This delay in release of funds had a negative effect on the implementation process of the plans as identified priorities could not be implemented at the appropriate time due to lack of funds when needed.

*“…when we do the budget like we are supposed to get this funding on quarterly basis but at times you can even get only once in a year*. *So you wait*, *wait*, *wait*, *wait…” C*ounty Manager 4, County B

#### Centralized management of county budgets

Across the three counties, the CDoH budgets were mainly managed centrally at county level and with very minimal operational resources being sent to facilities for addressing recurrent needs. Procurement of commodities and major renovations was all being handled at county level on behalf of the health facilities. This thus limited the ability health facility managers to implement their local (facility level) priorities at the appropriate time until at a time when these priorities are considered by the county managers as so.

*“In terms of financial resources*, *they have been mostly controlled at the county level*.*” County Manager 8*, *County A*

### Outcomes of devolved health sector planning and budgeting

#### Increase in health sector budgetary allocation within counties

**Fig 2:**
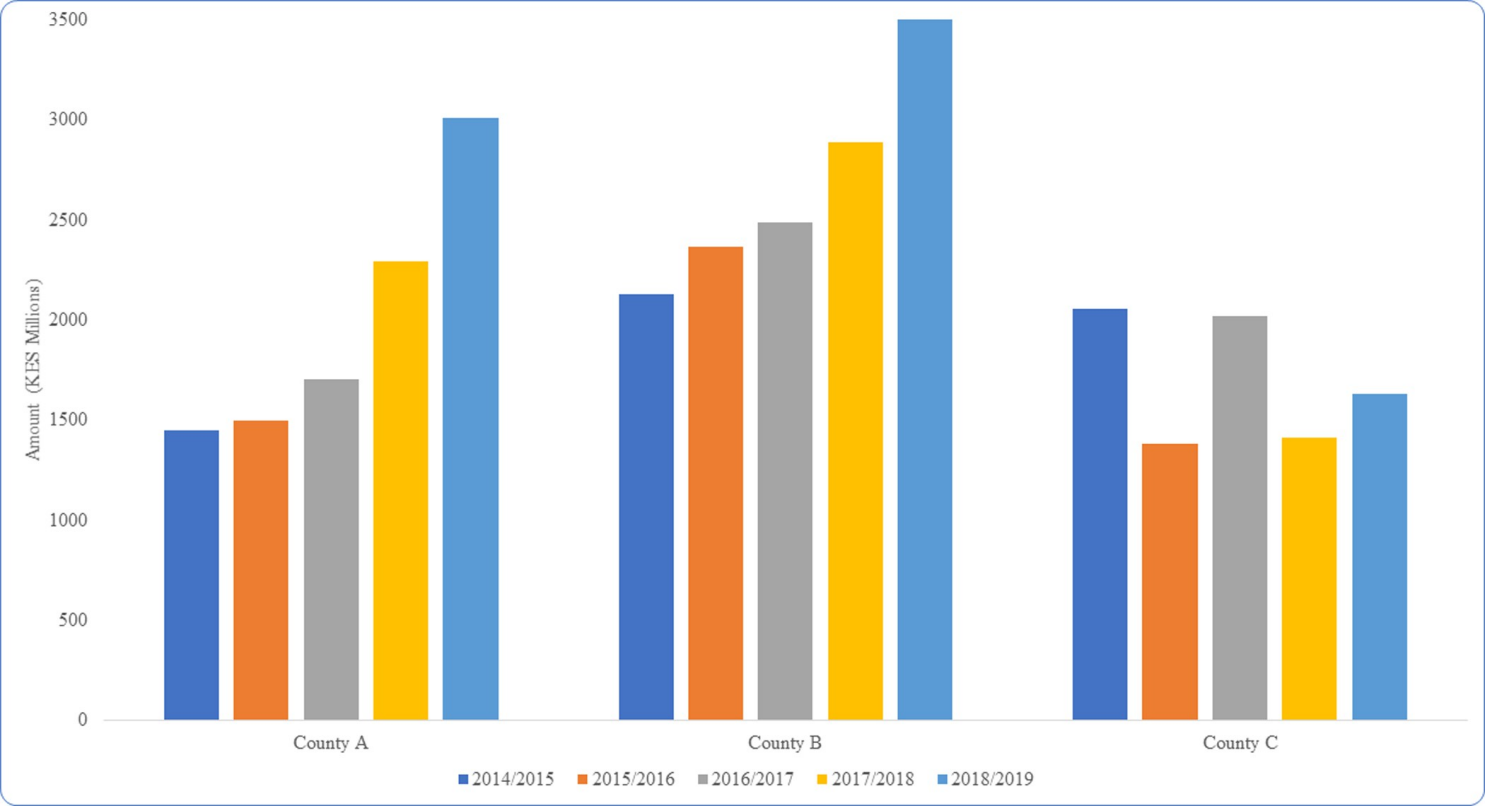
Trends for overall health sector budget allocation in study counties.

Fromm KIIs, respondents across the case study counties reported that there has been a progressive increase in health sector budget allocation within the counties since the advent of devolution. This was corroborated by our review of secondary data on the county government budget records. From this data, the progressive increase in health sector budgetary allocation was more marked in case study counties A and B, while case study county C had slight variation of health sector budget allocation patterns over the years. Fig 2 provides an illustration of trends in county level budgetary allocation across the three case study counties since devolution.

#### Coordinated support from county level partners

Even with the increase in county government’s allocation to health, all three counties reported to still have inadequate resources to meet CDoH needs. Interviewees reported that local level partners and donors have been helping to address some of the resource gaps. It was reported that due to devolution, counties now had power to directly engage with potential partners and funders to support county-level health sector priority activities. This signified the increase in decision space for health sector resource mobilisation occasioned by the devolution.

*“I don’t think there is any (budget) item I can say is fully*, *funded*. *But what is almost adequately funded especially–because there is support from our development partners–”* County Manager 5, County B

### Effects of COVID-19 on county health sector priority setting, planning, and budgeting

Across the counties, it was reported that the COVID-19 pandemic significantly altered health sector management and health service delivery. Health resources–including health managers and health workers–were reassigned to COVID-19 emergency response activities. This disrupted the focus from routine health management as well as affected routine health service delivery functions. Due to this, health service delivery functions such as immunization and maternal and child health services were disrupted significantly.

*“…*‥*Since the declaration of COVID–I think in March 2020 –we have diverted a lot of health resources*. *I think probably a hundred million in purchasing of PPEs and in employment of additional staff to manage COVID centers and in tracing … So a lot of resources have been diverted from what they would have done……”*. County Manager 2, County B

Similarly, management functions such as supervisory visits, management meetings by facility boards, public engagement in planning and budgeting, and stakeholder engagement in budget development were all disrupted by the COVID-19 pandemic.

In addition, the COVID-19 pandemic was reported to have affected public participation since public involvement now happened through mass media platforms such as radio and social media platforms, with information dissemination happening through community health assistants. This was felt to have reduced the effectiveness of public participation as comments made on social media were perceived to be overly critical and not actionable. It was also felt that COVID-19 limited public participation to those conversant with virtual technologies.

## Discusion

By applying political economy analysis which is an important but largely underutilised methodology in health systems and policy analysis research, our study adds new insights to the growing body of literature that has studied health sector priority setting, planning, and budgeting. The PEA is particularly important as it provide an opportunity to unpack contextually complex phenomena; and their effects and influences of the stated problem with a view of identifying possible pathways for potential solutions.

From our study, we found county-level health sector priority setting, planning, and budgeting are guided by elaborate legislation, policies and procedures that have been institutionalised since devolution. The devolution laws clearly outlined direct and indirect mechanisms of community and public participation in health sector priority setting and resource allocation processes. In addition, we found devolution to have increased the number of actors involved in county-level health sector priority setting, planning, and budgeting, many of whom have tense relationships owing to different interests and perspectives. Our study also found national health sector priority agenda issues, local political interest, equity considerations, and the relationships between various actors involved in the priority setting process to be driving county-level processes. In relation to accountability structures and processes, our study found that there are elaborate mechanisms for community and bureaucratic accountability in the counties. We further found that insufficient budget ceiling, rushed planning and budgeting process, erratic release of budgeted funds and centralised management of health sector budgets at county level are some of the challenges that have continued to affect both development and implementation of health sector priorities, plans and budgets. In addition, our study found that despite all these challenges, county governments have progressively increased health sector budgetary allocation since the advent of devolution. Counties have also, through devolution, gained the power to directly engage with potential donors and funders to support their health sector priorities.

Drawing from our conceptual framework, structural diagnosis focusses on ***structural features*** and ***rules of the game*** that oversee, coordinate, and facilitate a relevant policy issue. In relation to structural diagnosis, our study found that health sector priority setting, planning and budgeting processes in the three case study counties were guided by clear legal and policy procedures. These procedures cascade from national-level legal requirements to county governments for implementation, where counties have established county government structures that include a unit responsible for the coordination of planning and budgeting within the CDoH. These finding reveal progressive improvement and maturation of county government capacity for health sector planning and budgeting. This contrasts with previous studies which had reported structural capacity challenges in the counties in the early days of devolution [26, 27], when county governments were given the responsibility of undertaking health sector planning and budgeting before all the appropriate county government structures had been established [8, 15]. In addition, the action by the various county level actors to cascade and orient the lower level health facilities in the planning and budgeting process has been serving to strengthen the process.

Agency diagnosis focuses on ***actor*s**, ***motivations*,** and ***concepts*** around a policy issue. In relation to actors, our study found that devolution laws and other subsidiary legalisation that are in place increased the number of actors involved in county-level health sector priority setting, planning and budgeting. In addition, these laws elaborated the roles and mechanisms for community and public participation in health sector priority setting and resource allocation–both directly and through elected (political and facility committee) representatives. By expanding the space and role of communities and public participation in resource allocation planning and budgeting, Kenya’s devolution seems to be increasing the role of public participation in decision making. This is in tandem with historical arguments that have outlined the goals of public sector decentralisation policies [28–30]. The outcome of decentralisation policies to increase community and public participation in health sector planning and budgeting has also been reported in other countries in the region such as Uganda and Tanzania [30, 31]. In addition, our study reported that the relationship between the various actors involved in health sector planning and budgeting was tense. This does not come as a surprise considering the varied interests of different actors. Similar observations have been reported in Uganda and Tanzania before [30].

In relations to the drivers of the county level health sector priority setting, our study found that national-level health sector priority agenda tends to influence priority setting at the county level. However, increasingly, local level factors such as county-level political commitments, equity considerations, and local level actors’ relations are now increasingly influencing the priority setting process. This finding is in contrast to the health sector priority setting studies done prior to devolution in Kenya, which had found health sector priority setting, planning and budgeting to be heavily top-down [1, 3, 4]. The increase in local level drivers of health sector priority setting as a result of decentralisation has also been reported in health sector decentralisation studies in Uganda [30, 32].

Our study found devolution has strengthened the mechanisms for community and bureaucratic accountability in the study counties. Under the devolved government system in Kenya, public participation mechanisms were introduced to deliberately provide for public participation in the governance of public resources–including health sector resources [8, 33]. However, some of the county-level health managers felt that the involvement and participation of communities–either directly or indirectly through elected leaders–brought about politicization of the process and hence was not in the best interest of county-level health sector priority setting. Moreover, the growing influence of political appointees / representatives could be seen as displacing the voice of healthcare providers in health sector planning [34]. This could be an underlying contributor to the well-documented strikes in the health sector [35] and renewed efforts by providers to extend their influence into non-clinical policy arenas [36]. The contrasting views on the value and effects of public participation in governance of resources has been reported by Mitchel and Bossert, who in their 2010 analysis argued that the stakeholders views on the value of decentralisation normally depends on their positionality within the public sector service delivery [37]. Collins on his part argues that a clear understanding of the effects of stakeholders views on decentralisation calls for political and critical analysis [38].

From our conceptual framework, change focuses on *i****mplementation*** experiences and ***pathways to change*** and/or improvements of the policy issue. We found that insufficient budget ceiling, rushed planning and budgeting process, erratic release of budgeted funds, and centralised management of health sector budgets at county level are some of the challenges that have continued to affect development and implementation of health sector priority setting, planning and budgeting. Recentralised financial management under devolution had been reported in earlier studies on devolution in Kenya, and it seems to be a persistent and un-intended consequence of the devolution [39].

In addition, our study found that despite all these challenges, county governments have been progressively increasing health sector budgetary allocation since the advent of devolution. Increase in health sector budgetary allocation as a results of decentralisation has also been reported in Uganda [32]. Counties have also gained the power to directly engage with potential donors and funders to support their health sector priorities. This signifies the increase in county level decision space that was brought about by the devolved government system in the country [28].

## Policy implications

Our study findings present a couple of policy implications and recommendations. There is need and room to improve the sequence of decision making in planning and budgeting at the county level; by ensuring that respective county department budget ceilings are provided ahead of the development of departments Annual Work Plans. The health sector planning and budgeting process could also be improved through strengthening the capacity of the actors including CDoH managers and MCAs who have crucial roles in leading and overseeing the process.

Overall accountability for the process could be strengthened by strengthening the processes for public participation during the budget making process. This could be done by for example decentralisation the organisation of public participation forums to lower-level unit below the county. This will encourage attendance and participation of communities at village level. In addition, accountability can further be enhanced by strengthening the functioning of FMCs at facility level.

Finally continuous engagement and education of the various actors involved in the health sector planning and budgeting process to see their roles as complimentary will go in a long way to reduce the tense relationships of the different actors during the process.

## Study limitations

This study has several limitations relating to study design, implementation, and data availability. First, the larger project’s need for cross-country comparisons meant a more centralized strategy to the development of the study design, instruments, and analysis approach, which may have resulted in loss of context specificity in individual study countries. To address this, each country team refined study components to better capture local elements that influence management, governance, and decision-making. Second, the in-depth nature of data collection did not permit the inclusion of all DHSSi sites. Country teams selected specific sites to reflect critical features such as variability in geographic location, socio-economic contexts and other health system performance indicators. Third, due to the COVID-19 pandemic, all interviews were conducted remotely. We note that the impersonal nature of remote interviews made it impossible for the interviewers to pick up non-verbal cues during the interview. However, we tried to mitigate around data reliability by triangulating data from various sources.

## Conclusions

Our research shows that while devolution has greatly expanded community participation in county-level health sector priority setting, planning, and budgeting, it has also complicated and politicized decision-making. County governments now have the authority to allocate resources based on the preferences of their constituents by developing and executing AWPs. This process is often slow, noisy, and not straightforward despite improving accountability at the county level. Moreover, budgets are often not aligned with AWPs, are based largely on historical allocations, subject to delays from national government, undermined by inconsistent supply chains, and limited by the variable capacity of administrators. In summary, devolution has greatly transformed County health sector governance management in Kenya with longer-term potential for greater accountability and health equity, but with short-to-medium term challenges in developing efficient systems for engaging a diverse array of stakeholders in planning and budgeting processes.

## References

[1] Oyaya CO, Rifkin SB. Health sector reforms in Kenya: an examination of district level planning. Health Policy. 2003;64(1):113–127. doi: 10.1016/s0168-8510(02)00164-1 12644333

[2] Odhiambo T. Read Kenyan memoirs to understand devolution story. Published 2013. Accessed April 21, 2022. https://hakipensheni.blogspot.com/2013/06/read-kenyan-memoirs-to-understand.html

[3] O’Meara WP, Tsofa B, Molyneux S, Goodman C, McKenzie FE. Community and facility-level engagement in planning and budgeting for the government health sector—a district perspective from Kenya. Health Policy. 2011;99(3):234–243. doi: 10.1016/j.healthpol.2010.08.027 20888061 PMC4503225

[4] Tsofa B, Molyneux S, Goodman C. Health sector operational planning and budgeting processes in Kenya-"never the twain shall meet". Int J Health Plann Manage. 2016;31(3):260–276. doi: 10.1002/hpm.2286 25783862 PMC4988384

[5] Government of Kenya. Government Financial Management (Health Sector Services Fund) Regulations, 2007. http://kenyalaw.org/kl/index.php?id=649; 2007.

[6] Waweru E, Goodman C, Kedenge S, Tsofa B, Molyneux S. Tracking implementation and (un) intended consequences: a process evaluation of an innovative peripheral health facility financing mechanism in Kenya. Health Policy Plan. 2016;31(2):137–147. doi: 10.1093/heapol/czv030 25920355 PMC4748125

[7] Waweru E, Opwora A, Toda M, et al. Are Health Facility Management Committees in Kenya ready to implement financial management tasks: findings from a nationally representative survey. BMC Health Serv Res. 2013;13(1):1–14. doi: 10.1186/1472-6963-13-404 24107094 PMC3853226

[8] Tsofa B, Molyneux S, Gilson L, Goodman C. How does decentralisation affect health sector planning and financial management? a case study of early effects of devolution in Kilifi County, Kenya. Int J Equity Health. 2017;16(1):151. doi: 10.1186/s12939-017-0649-0 28911325 PMC5599897

[9] Njuguna DK, Wangia E, Wainaina S, Ndavi TW. Health Sector Planning at the County Level in Kenya: What has Worked, Challenges and Recommendations. Sci Acad Publ. 2020;10(3):87–93. doi: 10.5923/j.phr.20201003.01

[10] Waithaka D, Kagwanja N, Nzinga J, et al. Prolonged health worker strikes in Kenya- perspectives and experiences of frontline health managers and local communities in Kilifi County. Int J Equity Health. 2020;19(1):23. doi: 10.1186/s12939-020-1131-y 32041624 PMC7011250

[11] Tsofa B, Musotsi P, Kagwanja N, et al. Examining health sector application and utility of program-based budgeting: County level experiences in Kenya. Int J Health Plann Manage. 2021;36(5):1521–1532. doi: 10.1002/hpm.3174 33955046 PMC8519121

[12] Government of Kenya. The Constitution of Kenya: Distribution of functions between the national government and county governments. Fourth Schedule (Article 185 (2), 186 (1) and 187 (2)). Published online 2010.

[13] Tsofa BK. Examining the Effects of Political Decentralisation in Kenya on Health Sector Planning and Budgeting: A Case Study of Kilifi County.; 2017. doi: 10.17037/PUBS.03817566

[14] Tsofa B, Goodman C, Gilson L, Molyneux S. Devolution and its effects on health workforce and commodities management—early implementation experiences in Kilifi County, Kenya. Int J Equity Health. 2017;16(1):169. doi: 10.1186/s12939-017-0663-2 28911328 PMC5599882

[15] Waithaka D, Tsofa B, Kabia E, Barasa E. Describing and evaluating healthcare priority setting practices at the county level in Kenya. Int J Health Plann Manage. Published online April 2018. doi: 10.1002/hpm.2527 29658138 PMC6120533

[16] Tama E, Molyneux S, Waweru E, Tsofa B, Chuma J, Barasa E. Examining the Implementation of the Free Maternity Services Policy in Kenya: A Mixed Methods Process Evaluation. Int J Heal policy Manag. 2017;7(7):603–613. doi: 10.15171/ijhpm.2017.135 29996580 PMC6037504

[17] Mwakisha JW. Keeping to the universal health coverage path in Kenya. WHO Regional Office for Africa. Published 2020. Accessed April 24, 2022. https://www.afro.who.int/news/keeping-universal-health-coverage-path-kenya

[18] Kenya Vision 2030. County Governments At The Centre of Achieving Universal Health Care | Kenya Vision 2030. Kenya Vision 2030. Accessed April 24, 2022. https://vision2030.go.ke/county-governments-at-the-centre-of-achieving-universal-health-care/

[19] Tsofa B, Munywoki J, Waweru E, Koon A. Political Economy Analysis of Sub-National Health Management in Eastern and Southern Africa: UNICEF Kenya Project Report, Final Draft.; 2021.

[20] United Nations Children’s Education Fund. The UNICEF Health Systems Strengthening Approach.; 2016. https://www.unicef.org/media/60296/file

[21] George A, Ng C, Coelho VS, Okungu V, Bennett S. Exploring how political economy analysis is understood by health policy and system researchers researchers—SHaPeS crowd-sourcing collaboration. Published online 2014. https://www.researchgate.net/profile/Vincent-Okungu/publication/314081906_Exploring_how_political_economy_analysis_is_understood_by_health_policy_and_system_researchers_SHaPeS_crowd-sourcing_collaboration/links/58b3f88a45851503be9e21e2/Exploring-how-polit

[22] United Kingdom -Department for International Development. Political Economy Analysis How To Note—A DFID Practice Paper. UK-DFID; 2009.

[23] Fritz V, Levy B, Ort R. Problem-Driven Political Economy Analysis: The World Bank’s Experience. International Bank for Reconstruction and Development/The World Bank; 2014. doi: 10.1596/97i-1-4648-0121-1

[24] Siddiqi S, Masud TI, Nishtar S, et al. Framework for assessing governance of the health system in developing countries: gateway to good governance. Health Policy (New York). 2009;90(1):13–25. doi: 10.1016/j.healthpol.2008.08.005 18838188

[25] Ministry of Health. Kenya Health Sector Partnership and Coordination Framework (2018–2030). Published online 2017. https://www.health.go.ke/wp-content/uploads/2021/02/KENYA-Health-R-B5-1_1-15.pdf

[26] Nyikuri MM, Tsofa B, Okoth P, Barasa EW, Molyneux S. “We are toothless and hanging, but optimistic”: sub county managers’ experiences of rapid devolution in coastal Kenya. Int J Equity Health. 2017;16(1):113. doi: 10.1186/s12939-017-0607-x 28911332 PMC5599878

[27] Nyikuri M, Tsofa B, Barasa E, Okoth P, Molyneux S. Crises and Resilience at the Frontline-Public Health Facility Managers under Devolution in a Sub-County on the Kenyan Coast. PLoS One. 2015;10(12):e0144768. doi: 10.1371/journal.pone.0144768 26696096 PMC4687913

[28] Bossert T. Analyzing the decentralization of health systems in developing countries: decision space, innovation and performance. Soc Sci Med. 1998;47(10):1513–1527. doi: 10.1016/s0277-9536(98)00234-2 9823047

[29] Bossert TJ, Beauvais JC. Decentralization of health systems in Ghana, Zambia, Uganda and the Philippines: a comparative analysis of decision space. Health Policy Plan. 2002;17(1):14–31. doi: 10.1093/heapol/17.1.14 11861583

[30] Kapiriri L, Norheim OF, Heggenhougen K. Public participation in health planning and priority setting at the district level in Uganda. Health Policy Plan. 2003;18(2):205–213. doi: 10.1093/heapol/czg025 12740325

[31] Shayo EH, Mboera LEG, Blystad A. Stakeholders’ participation in planning and priority setting in the context of a decentralised health care system: the case of prevention of mother to child transmission of HIV programme in Tanzania. BMC Health Serv Res. 2013;13:273. doi: 10.1186/1472-6963-13-273 23849730 PMC3720200

[32] Jeppsson A. Financial priorities under decentralization in Uganda. Health Policy Plan. 2001;16(2):187–192. doi: 10.1093/heapol/16.2.187 11358920

[33] McCollum R, Taegtmeyer M, Otiso L, et al. “Sometimes it is difficult for us to stand up and change this”: an analysis of power within priority-setting for health following devolution in Kenya. BMC Health Serv Res. 2018;18(1):906. doi: 10.1186/s12913-018-3706-5 30486867 PMC6264027

[34] McCollum R, Theobald S, Otiso L, et al. Priority setting for health in the context of devolution in Kenya: implications for health equity and community-based primary care. Health Policy Plan. 2018;33(6):729–742. doi: 10.1093/heapol/czy043 29846599 PMC6005116

[35] Irimu G, Ogero M, Mbevi G, et al. Tackling health professionals’ strikes: an essential part of health system strengthening in Kenya. BMJ Glob Heal. 2018;3(6):e001136. doi: 10.1136/bmjgh-2018-001136 30588346 PMC6278918

[36] Koon AD. When Doctors strike: Making Sense of Professional Organizing in Kenya. J Health Polit Policy Law. 2021;46(4):653–676. doi: 10.1215/03616878-8970867 33493308

[37] Mitchell A, Bossert TJ. Decentralisation, governance and health‐system performance:‘where you stand depends on where you sit.’ Dev Policy Rev. 2010;28(6):669–691.

[38] Collins C. Decentralization and the need for political and critical analysis. Health Policy Plan. 1989;4(2):168–171.

[39] Barasa EW, Manyara AM, Molyneux S, Tsofa B. Recentralization within decentralization: County hospital autonomy under devolution in Kenya. PLoS One. 2017;12(8):e0182440. doi: 10.1371/journal.pone.0182440 28771558 PMC5542634

